# Transcriptome Signatures of Canine Mammary Gland Tumors and Its Comparison to Human Breast Cancers

**DOI:** 10.3390/cancers10090317

**Published:** 2018-09-07

**Authors:** Kang-Hoon Lee, Hyoung-Min Park, Keun-Hong Son, Tae-Jin Shin, Je-Yoel Cho

**Affiliations:** 1 Department of Biochemistry, BK21 Plus and Research Institute for Veterinary Science, School of Veterinary Medicine, Seoul National University, Seoul 08826, Korea; khlee02@snu.ac.kr (K.-H.L.); phm0525@snu.ac.kr (H.-M.P.); taejin430@snu.ac.kr (T.-J.S.); 2 Department of Microbiology, College of Natural Sciences, Dankook University, Cheonan 31116, Korea; 52101358@dankook.ac.kr

**Keywords:** transcriptome, dog, mammary gland tumor, breast cancer, RNA-seq

## Abstract

Breast cancer (BC)/mammary gland carcinoma (MGC) is the most frequently diagnosed and leading cause of cancer-related mortality in both women and canines. To better understand both canine MGC and human BC-specific genes, we sequenced RNAs obtained from eight pairs of carcinomas and adjacent normal tissues in dogs. By comprehensive transcriptome analysis, 351 differentially expressed genes (DEGs) were identified in overall canine MGCs. Based on the DEGs, comparative analysis revealed correlation existing among the three histological subtypes of canine MGC (ductal, simple, and complex) and four molecular subtypes of human BC (HER2+, ER+, ER&HER2+, and TNBC). Eight DEGs shared by all three subtypes of canine MGCs had been previously reported as cancer-associated genes in human studies. Gene ontology and pathway analyses using the identified DEGs revealed that the biological processes of cell proliferation, adhesion, and inflammatory responses are enriched in up-regulated MGC DEGs. In contrast, fatty acid homeostasis and transcription regulation involved in cell fate commitment were down-regulated in MGC DEGs. Moreover, correlations are demonstrated between upstream promoter transcripts and DEGs. Canine MGC- and subtype-enriched gene expression allows us to better understand both human BC and canine MGC, yielding new insight into the development of biomarkers and targets for both diseases.

## 1. Introduction

Human breast cancer (BC) is one of the most common cancers in women and is a leading cause of death worldwide, accounting for 8.8 million deaths in 2015 [1]. Approximately 80% of diagnosed BCs are invasive and heterogeneous, consisting of up to 21 distinct histological subtypes [2]. Current biological markers used for evaluating molecular subtypes of BC include hormone receptors for estrogen or progesterone, and HER2+/−, indicating levels of human epidermal growth factor receptor 2 (HER2) [3]. Although large-scale cohort studies using gene expression profiling techniques, such as next-generation sequencing, have provided better understanding of the molecular regulation of BC, a limited number of studies have been performed in rare and aggressive subtypes of human BC, such as invasive ductal carcinoma, myoepithelial complex type BC, and inflammatory BC [4,5,6].

Canine mammary gland carcinoma (MGC) is a well-known animal model for human BC, as there are a number of benefits to studying human BC using dogs [7]. Existing similarities between these species have been reported with respect to genetic, biological, anatomic, and clinical features [8,9]. Additionally, dogs hold a unique status in human BC studies with respect to epigenetic aberrations since both dogs and humans, especially companion dogs and owners, share neighborhood environments and might be exposed to the same carcinogens [10]. Moreover, in contrast to human BC, complex/mixed MGC consisting of epithelial masses containing regions of myoepithelial components comprises the majority of MGC in dogs [11]. Thus, since the dog reference genome was unveiled in 2005, a number of comparative analyses using transcriptome data in independent studies have been performed [12,13,14]. However, the results of these studies have been relatively inconsistent and only few biomarkers have been identified for canine MGC as well as human BC [15,16].

In the last few decades, high-throughput sequencing technology in medical oncology has generated a large number of databases including genetic mutations, gene expression profiles, and epigenetic aberrations associated with diverse cancer types [17,18]. Many gene expression profiling studies on human BC carcinogenesis have also been performed with large BC patient cohorts and have reported many differentially expressed genes (DEGs) and their related cancer pathways [19,20].

Noncoding RNAs (ncRNAs) have become one of the most highlighted transcriptomic features in diverse organisms, increasing our understanding of the complexity of transcriptomic regulation. More than several tens of thousands of ncRNAs have been identified and have been functionally grouped within human and model organisms, such as yeast and mouse [21,22,23]. Particularly in human BC, a list of microRNAs (miRNAs) are considered to have crucial roles in cancer development and metastasis, and other studies have shown that miRNA expression profiles of each breast cancer subtype are different [24,25]. Moreover, a cluster of oncogenic long ncRNAs (lncRNA) are up-regulated in human BC and seem to be involved in regulating immune system activation [26]. Additional interesting ncRNAs, including those recently determined and confirmed in existence, are known as promoter upstream transcripts (PROMPTs) [27]. Interestingly, the presence of PROMPTs may be positively correlated with gene activity. Although PROMPTs are not widespread regulators of gene expression, their existence is tightly regulated by exosome activity, and the analysis of PROMPTs as a part of regulatory mechanisms of transcription in cancer might be important to better understand MGC.

In this study, we sequenced total RNAs from 10 pairs of canine MGC and matching adjacent normal tissues to identify canine MGC-associated transcriptomic signatures. We further tested whether these signatures can distinguish canine MGCs from normal tissue using principal component analysis (PCA) and clustering. To better understand both canine MGCs and human BC, we subsequently extracted a group of canine MGC-associated KEGG pathways and gene ontology (GO) terms. PROMPTs were then suggested as a part of transcriptional regulation mechanisms in cancer. This study will provide new insights into biomarker and target development for human BC as well as canine MGC.

## 2. Results

### 2.1. RNA Sequencing in Mammary Gland Tumors and Matching Adjacent Normal Dog Tissues

Ten dogs with mammary gland tumors (MGT) were enrolled in this study as well as pairs of MGCs, and matching adjacent normal tissues were collected by veterinarians during surgery and pathologically tested. Animal protocols were approved by SNU IACUC (approval#SNU-170602-1, 26 July 2016). Out of ten dogs, two dogs were excluded from this study due to diagnosis of benign adenoma and large differences in the phylogenetic tree of dog breeds. To increase the reliability of the RNA sequencing data, each subtype consists of at least two specimens as biological replicates (three specimens in ductal, three in complex, and two in simple type). Ultimately, eight pairs of data of MGC and normal tissues were further analyzed (Table S1).

**Figure 1:**
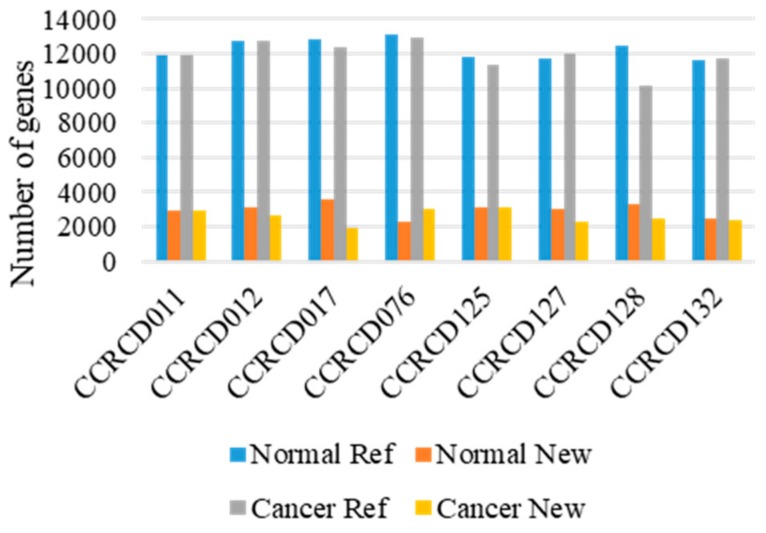
Transcript expression found in 8 pairs of mammary gland tumors (MGTs) and matching adjacent normal tissues. Ref: Canfam3.1 reference annotation.

Overall, 625.4 and 672.4 million paired-end and strand-specific reads from dog MGC and adjacent normal tissues were sequenced, respectively (Table S2). The transcript integrity number (TIN) was computed to measure RNA degradation level (Table S3). Both raw read quality scores (Q30) and median TINs for all the samples were greater than 93.17% and 65%, respectively. Before sequence alignment, gene transfer format (GTF) of Canfam3.1 reference annotation file was updated with our dog transcript library consisting of 10,792 novel transcripts with information obtained from 13 major dog organs (unpublished data, manuscript in preparation). Out of 1.29 billion reads, more than 96.82% reads were mapped onto Canfam3.1, the canine reference genome reinforced by our annotation file. Unique transcripts where the regions had never been annotated in dog were considered “novel”. Overall, in a total of 8 pairs of transcriptome, the number of transcripts identified with both novel and reference annotations were slightly higher in the adjacent normal tissues (5015 new and 15,602 ref genes) than in the MGC tissues (4683 new and 15,003 ref genes) (Figure 1 and Table S4). 

### 2.2. Identification of DEGs in Canine MGTs and Their Subtypes

**Figure 2:**
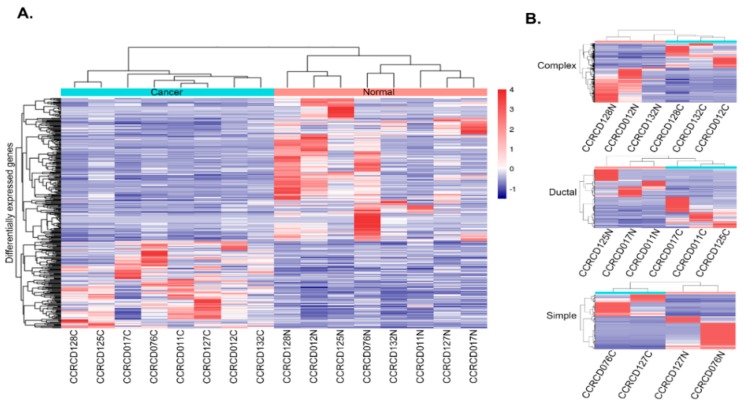
Heat map and hierarchical clustering of mammary gland carcinoma (MGCs) and matching adjacent normal tissues. (**A**) in eight pairs and (**B**) in three subtypes of MGCs (complex, simple, and ductal). Eight specimens were labeled with N (normal) and C (cancer). The distance metric used for clustering was Kendall correlation, while the linkage method used was average linkage.

For the differentially expressed gene (DEG) analysis, four comparisons were performed between eight pairs of MGCs and matching adjacent normal tissues and in three subtypes (simple, complex and ductal). DEGs with a *p*-value < 0.01 and changes greater than 2-fold were determined for each comparison. Cuffdiff analysis identified 350 DEGs, of which 132 and 218 genes were up- and down-regulated, respectively, in a comparison of the eight canine MGCs and matching adjacent normal tissues (Table S5). Hierarchical clustering with Kendall correlation matrix of the 350 DEGs successfully distinguished MGCs and matching adjacent normal in a heat map analysis (Figure 2A). In total, 454 DEGs (178 up- and 276 down-regulated), 226 DEGs (117 up- and 109 down-regulated) and 171 DEGs (66 up- and 105 down-regulated) were identified as subtype-specific DEGs for complex, ductal, and simple MGCs respectively. Hierarchical clustering with these DEGs successfully separated MGC from normal again (Figure 2B). 

**Figure 3:**
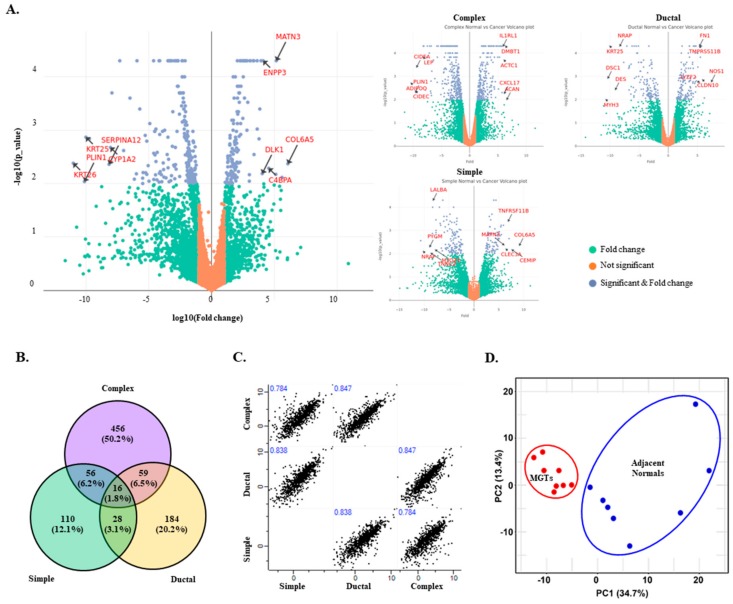
Differentially expressed genes (DEGs) in canine MGCs. (**A**) Volcano plots of DEG content with larger than two-fold changes (log 2 values) and *p*-values < 0.001 for each comparison. (**B**) Venn diagrams illustrating the number of up- and down-regulated DEGs among three subtypes of MGC. (**C**) Scatter plots of DEGs among three subtypes of MGC. The Spearman rank correlation based on 555 DEGs was computed by Perseus (ver.1.5.8.5) in Maxquant software. (**D**) Principal Component Analysis (PCA). The first three principal components explain ~57% of total variations.

Overall DEGs were summarized and visualized using Venn diagram and Volcano plots (Figure 3). The top five up-/down-DEGs were labeled in Volcano plots (Figure 3A) and are listed in Table 1. Out of 851 DEGs, only 16 genes, 1.6% of total DEGs, were shared by all three subtypes, indicating that these three subtypes might have unique RNA expression profiles (Figure 3B). Subsequently, correlations among DEG profiles in these three subtypes were tested and are shown in scatter plots (Figure 3C). All correlation coefficients among subtypes of MGC were between 0.7~0.9, which can be considered highly correlated. There was little difference between the highest correlation (0.849 between ductal and complex subtype) and the lowest correlation (0.784 between simple and complex subtype). Thus, each subtype of MGC had unique transcription signatures, but overall transcriptome profiles might be very similar among MGCs.

To test whether these DEG signatures represent canine MGCs and/or MGC subtypes, we conducted a principal component analysis (PCA). PCA results indicated that the first principal component (PC1) explains 34.7% of the variability, while PC2 and PC3 explain 13.4% and 10.6% of the variability, respectively, in DEGs of all the canine samples. Three PCs only covered ~58% of total variability. This might represent the complexity of cancer biology in clinical samples. Although these three PCs only covered approximately 58.7% of the total variability in the overall comparison of MGC and the adjacent normal tissue, MGC and the matching normal samples were successfully distinguished from each other in dimensional PCA, illustrated in Figure 3D. Unexpectedly, all eight MGCs were tightly grouped, whereas matching normal tissues were more individually variable (Figure 3D). 

### 2.3. Correlation in Gene Expression between Four Molecular Subtypes of Human BC and Three Histological Subtypes of Canine MGC

**Figure 4:**
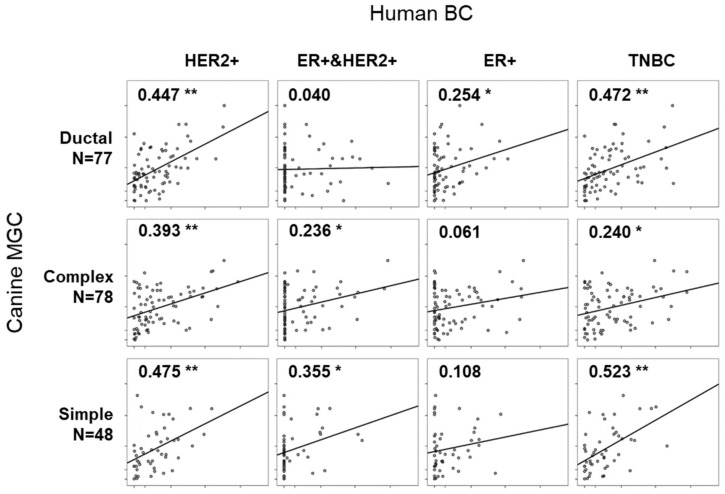
Scatter plots showing the correlation between molecular subtypes of human breast cancers (BCs) and histological subtypes of canine MGCs. Different numbers of canine MGC subtypes-specific genes were abstracted (Complex: N = 78, Ductal: N = 77, and Simple: N = 48). *, ** indicates *p* < 0.05, *p* < 0.01, respectively.

Eleven RNA-sequencing data for four molecular subtypes (HER2+, ER+, ER&HER2+, and TNBC) were retrieved from the study by Chung W. et al., publicly opened project (PRJNA305054) in the National Center for Biotechnology Information (NCBI) [28]. DEGs specific to each canine MGC subtype were subjected for correlation analysis (Table S6A). BC with molecular subtype of HER2+ showed significant correlation coefficient (r) with all three canine MGC subtypes (max r = 0.475 with simple subtype, min r = 0.393 with complex subtype, *p* < 0.01) (Figure 4). ER+ and ER+&HER2+ subtypes showed no correlation with ‘complex and simple’ and ductal subtype, respectively. Only low levels of correlation were found in ER+ with ductal subtype (r = 0.254, *p* < 0.05) and ER+&HER2+ with simple subtype (r = 0.355, *p* < 0.05). Notably, TNBC has strong correlation in both ductal and simple subtypes (r = 0.472 and 0.523, respectively). It is interesting because TNBC is usually defined as basal-like and non-basal-like types in human BC and the most common histological subtype of TNBC is invasive ductal carcinoma. Moreover, the simple subtype showing the highest correlation in TNBC expressed KRT5 and MKI67, which has been known and used as immunohistochemical markers for basal-like breast cancer and proliferation [29]. Our results indicated that transcriptomic signatures for canine MGC subtypes might represent human BC subtypes and provide new candidates of biomarkers. We then tested the same analysis oppositely using the gene expression profiles listed in PAM50 and Oncotype DX, but no significant correlation was found among subtypes of human BC and canine MGC (Table S6B).

### 2.4. Gene Ontology (GO) and Network Analysis

To better understand transcriptomic regulation in canine MGCs, we performed GO analysis with DEGs in all MGCs and in each subtype. For GO analysis, only the list of DEGs annotated by Ensembl gene name were subjected to ClueGo software (ver.2.5.0). Three hundred fifteen out of 350 profiled DEGs were assigned to 88 GO terms, including 53 biological processes (BP), 18 cellular components, and 18 molecular function terms. GO terms were mainly categorized into BPs with wide distributions and extensive assignments (53 GO terms). BP assignments in up-regulated DEGs in MGCs were divided into eight groups.

**Figure 5:**
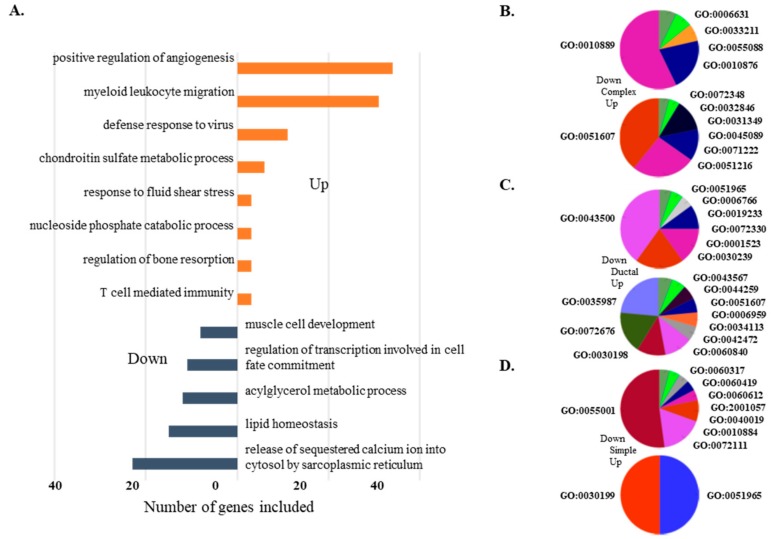
Gene ontology (GO) enrichment analysis for DEGs identified in an MGC-specific and subtype-dependent manner. (**A**) GO analysis using DEGs from all three subtype comparisons. Orange bar indicates up-regulated GO and dark blue bar represents down-regulated GO. GOID enriched in each comparison of (**B**) Complex type, (**C**) Ductal type, and (**D**) Simple type of MGT.

The most prevalent BP group, consisting of eight GO terms, was represented by positive regulation of angiogenesis (GO:0045766). This group also included some important assignments, such as “cell adhesion mediated by integrin (GO:0033627)” and “positive regulation of vasculature development (GO:1904018),” suggesting that the biological processes in MGCs were directionally changed to promote tumor progression with increased vasculature [30]. In contrast, the GO term “release of sequestered calcium ion into cytosol by sarcoplasmic reticulum” (GO:001480) represented BP in down-regulated DEGs. This result is interesting because association between calcium ion homeostasis and cancerization has been reported [31]. This group consisted of 5 GO terms (GO:0003009, GO:0003009, GO:0055002, GO:0048747 and GO:0055008) covering 33.3% of total GO terms in down-regulated DEGs (Figure 5A) (Table 2).

**Figure 6:**
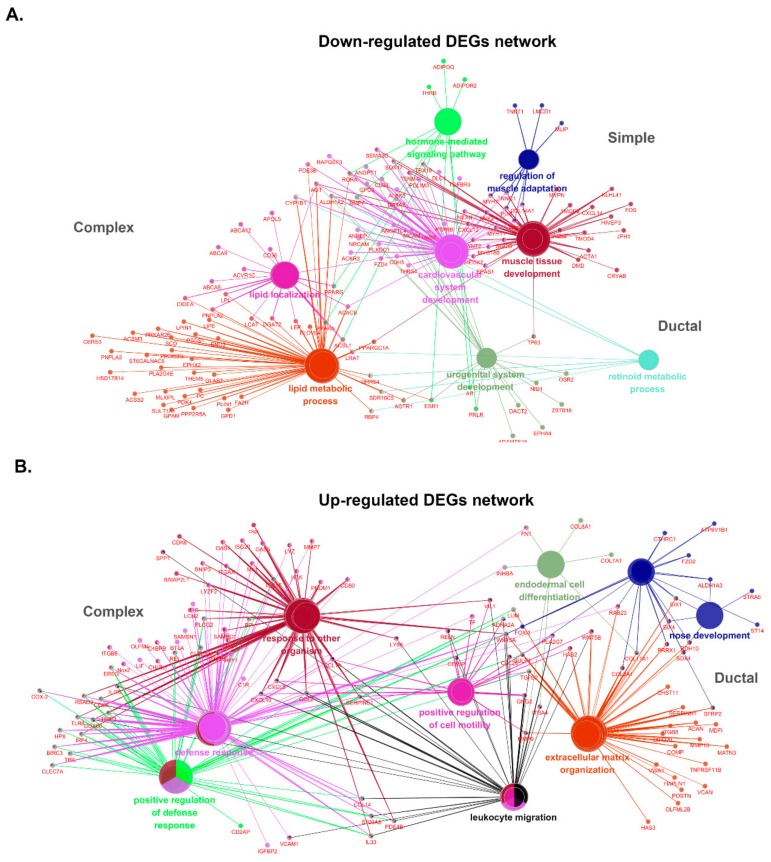
Gene network enrichment analysis in three subtypes of MGCs. (**A**) Down-regulated DEGs. Lipid metabolism and localization are enriched only in the complex subtype, while muscle-related biological processes are enriched in the ductal subtype. The simple subtype does not construct unique nodes. (**B**) Up-regulated DEGs. Response to other organisms and defense responses are highlighted in the complex subtype, but cell mobility and extracellular matrix organization are shown in the ductal subtype. No node was found up-regulated in the simple subtype.

Similar analyses were performed for DEGs within the three subtypes, and the data are shown in Table S7. The most prevalent group of BPs in up-regulated genes of the complex subtype is defense response to virus, covering 43.7% of up-regulated DEGs. Furthermore, some important assignments, such as cartilage development (GO:0051216), showed ~28.9%. Interestingly, 14 GO terms obtained from down-regulated DEGs in the complex subtype are grouped into five GO groups associated with lipid-related biological process, such as GO:0010876 that describes lipid localization, GO:0006631 of fatty acid metabolic process, and GO:0033211 of adiponectin-activated signaling. These results indicated the reduction of adipose components in the complex subtype compared to normal tissues. GO terms of defense response to virus (GO:0051607), humoral immune response (GO:0006959), and extracellular matrix organization (GO:0030199) up-regulated in the complex subtype were also shared by GO terms in the ductal subtype (Figure 5B). However, endoderm-related biological processes, such as endodermal cell differentiation (GO:0035987), endoderm formation (GO:0001706), primary germ layer formation (GO:0001704), and endoderm development (GO:0007492), were enriched only in the ductal subtype. Whereas lipid-related BPs were down-regulated in the complex subtype, many GO terms linked to muscles, such as cardiac muscle tissue morphogenesis (GO:0055008), skeletal muscle adaptation (GO:0043501), and muscle adaptation (GO:0043500), were found in down-regulated DEGs in the ductal subtype (Figure 5C). These down-regulated data suggested the dominant origin of ductal epithelium in ductal carcinoma compared to the presence of a certain proportion of myoepithelial cells in normal tissues. Since the sample numbers were relatively small in the simple subtype, only a few GO terms were identified as up-regulated (GO:0030199, GO:0051965). Numbers of GO terms enriched in down-regulated DEGs in the simple subtype were shared by one from the ductal subtype. Various muscle-related biological processes were also down-regulated (GO:0043500. GO:0035994, GO: 0048011, GO:0014888, GO:0055001, and GO:0055008) in simple carcinoma (Figure 5D). Gene networks constructed by DEGs enriched in canine MGCs are shown in Figure 6. 

### 2.5. Pathways Significantly Enriched in MGC

Many cancer-related pathways including WNT, PI3K/Akt, KRAS, and PTEN pathways have been reported in canines [32,33,34]. To better understand canine MGC and human BC, we performed KEGG pathway analysis using the web-based DAVID functional annotation tool (https://david.ncifcrf.gov/summary.jsp). For the pathway analysis, we used a list of DEGs summed by the three subtype comparisons because it showed better results than with DEGs from the overall MGC comparison. Out of 727 DEGs, 313 up- and 414 down-regulated DEGs in MGCs were isolated and subjected to KEGG pathway analysis (Table S8). Three hundred thirteen up-regulated DEGs in MGCs were involved in 24 and 23 KEGG pathways in dog and human databases, respectively. Twenty-one terms from the KEGG pathway analysis, including ‘ECM-receptor interaction’, ‘pathways in cancer’, and ‘proteoglycan in cancer’, were shared by both dog and human databases. However, the terms ‘microRNA in cancer’, ‘salivary secretion’, and ‘Wnt signaling pathway’ were found only in the dog database, while ‘dilated cardiomyopathy’ and ‘Fc gamma R-mediated phagocytosis’ were exclusively found only in the human database. The highest assignment of up-regulated DEGs was ‘pathways in cancer’ which includes WNT and PI3K pathways (Figure S1A). Seventeen up-regulated DEGs in canine MGC primarily mapped to ECM-ITGA/B-PI3K signaling and Wnt-Frizzled signaling pathways. ECM signaling is known to be involved in proliferation, migration, invasion, and angiogenesis [35]. In addition, up-regulation of COX2, TGFb, Glut1, MMP, and IL8 genes were involved in angiogenesis, and BIRC7/2 is known for its function of apoptosis evasion [36]. In contrast, ‘metabolic pathways’ was the highest enriched (45 genes) KEGG pathway among down-regulated DEGs. Interestingly, most DEGs were heavily mapped to glycan biosynthesis and metabolism, and some additionally mapped to lipid metabolism related to glycan biosynthesis and metabolism pathways (Figure S1B). These results indicated that aberration of lipid biogenesis and metabolism is associated with canine MGC progression. 

### 2.6. Accumulation of Promoter Upstream Transcripts (PROMPTs) and MGC-Associated Gene Transcription

**Figure 7:**
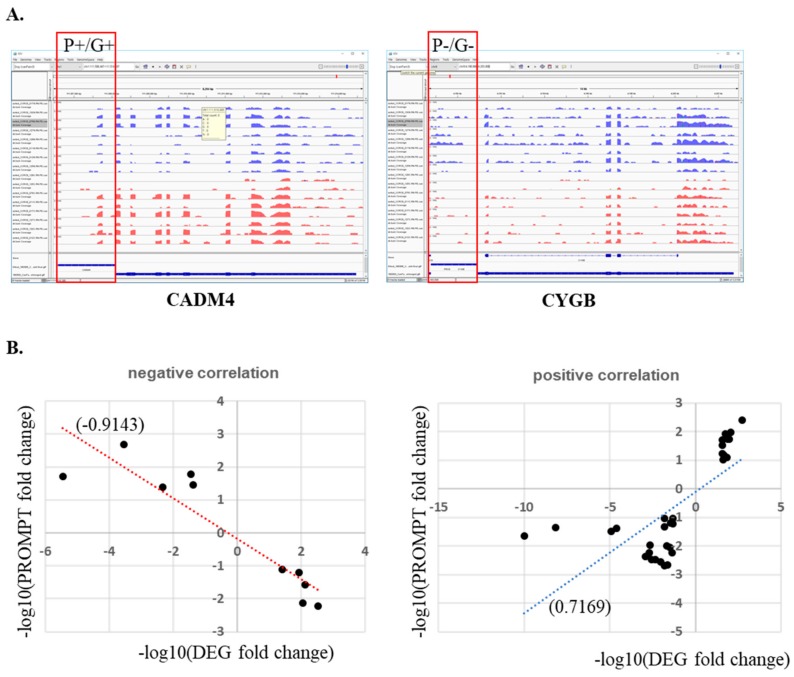
Correlation between DEGs and promoter upstream transcripts (PROMPT) expression. (**A**) CADM4 and CYGB gene promoter regions as an example of DEGs and PROMPT expression in integrative genomic viewer (IGV). (**B**) Negative and positive correlation between DEGs and PROMPTs.

Although some regulatory mechanisms have been suggested, few promoter upstream transcripts (PROMPTs) have been characterized, and many of their functional roles remain unknown [27]. Here, we measured unknown genome-wide transcripts expressed in the upstream regions of gene promoters. To quantify transcripts upstream of promoter regions, we collected all sequence reads mapped to regions ranging from all genes’ TSS to −1500 upstream. After excluding mapped transcript sequences that are shared with other genes, 28,757 promoter upstream regions consisting of 25,395 Ensembl database genes and 3362 novel transcripts were identified and used for further analysis. These were narrowed down to 41 regions (31 positive and 10 negative correlations) that met the threshold (*p* < 0.01, fold change ≥2) for genes and (fold change ≥ 2) PROMPTs. Unfortunately, differences in all ten negatively correlated genes and PROMPTs listed in Table S9 were not confirmed by integrative genomic viewer (IGV) due to low expression level of the transcripts. However, the genes and PROMPTs that were positively correlated were confirmed by IGV survey (correlation: 0.71694) (Figure 7). Eleven genes out of 31 were up-regulated in MGCs and positively correlated with PROMPT expression. Some of these promoter regions, such as NOVA1 and GRIA3, have been annotated with antisense RNA and pseudogenes, but most were not. This meant that more comprehensive genome annotations are necessary for the dog genome. Furthermore, it might provide a clue for understanding the regulatory mechanisms of up-regulated gene expression in cancer.

### 2.7. Quantitative Real-Time RT-PCR Validation of DEGs in MGTs

**Figure 8:**
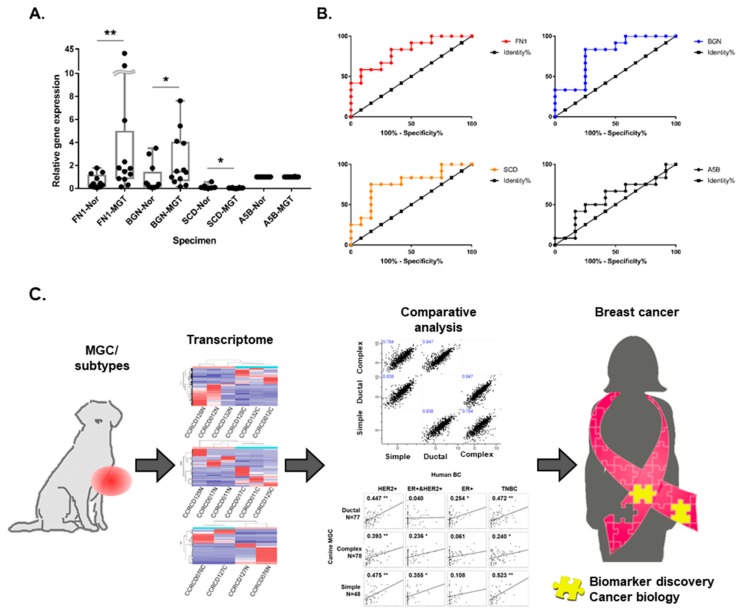
Real-time PCR for validation of MGC-enrichd RNA expression. (**A**) Box-and-whisker plots of relative gene expression levels in MGCs and matching adjacent normal samples. The Mann–Whitney test was performed. Bar graphs representing relative RNA expression of FN1, BGN, and SCD genes in 12 MGCs and adjacent normal tissues. Statistical significance is indicated by asterisks and relative *p*-value (** *p* < 0.01, * *p* < 0.05) (**B**) Receiver operating characteristic (ROC) curve for each gene expression level. (**C**) Conceptional scheme of canine MGC as a model to study human BC and discovery new biomarkers.

To validate our results, quantitative real-time RT-PCR was performed on ten selected DEGs to confirm our RNA-seq data. Out of ten, three well-verified genes were selected for further validation. The three genes belong to divergent functional categories or pathways but are not included in either Mammaprint or OncotypDx. FN1 (fibronectin 1) is involved in cell adhesion and migration. BGN (biglycan) plays a role in collagen fibril assembly in multiple tissues. SCD (stearoyl-CoA desaturase) belongs to the fatty acid desaturase family and is involved in fatty acid biosynthesis. Verification was performed in additional pairs of ten MGTs and matching adjacent normal samples using real-time RT-PCR. The relative gene expression to ATP5B gene was calculated by the 2^−ΔΔCt^ method and is shown in Figure 8. Up- or down-regulated MGC DEGs in RNA sequencing data were confirmed in most sample pairs. Up-regulated FN1 and BGN were validated in seven out of eight MGCs and matching normal tissues, respectively. In contrast, down-regulated SCD was confirmed in six out of eight MGCs (Figure 8A). The Mann–Whitney U test indicated that there was significant difference in gene expression levels between MGCs and adjacent normal tissues (FN1; U = 27, *p* = 0.0083, BGN; U = 31, *p* = 0.0173, SCD; U = 34, *p* = 0.0284). To expand this analysis, we performed a receiver operating characteristics (ROC) analysis for each gene (Figure 8B). A maximum AUC of 0.8125 (95% CI 0.6424–0.9826) was observed in FN1 gene expression. AUCs of 0.7847 and 0.7639 were observed for BGN and SCD, respectively.

## 3. Discussion

In this study, we performed genome-wide transcriptome analysis of spontaneous canine MGCs and compared to transcriptome data from four molecular subtypes of human BC. Although the sample size was small, being a pilot study (16 transcriptomes; 8 MGCs with matching adjacent normal tissues), this study could reveal transcriptome signatures enriched in canine MGC and subtypes.

Although there were several reports presenting that canine MGC is a good model for human BC study, subtype levels were still unclear [37,38]. We thus determined whether these two systems are compatible at the transcriptome level. We analyzed correlation in gene expression existing between the subtypes of human BC and canine MGC using the genes differentially expressed in canine MGC. Overall, the level of correlation seemed low between human BC and canine MGC (max r = 0.523, min r = 0.040). However, correlation among the subtypes within canine MGC was not strong either (max r = 0.767) (Table S6B). It means that each subtype of canine MGC has a unique gene expression pattern. One of the interesting findings in the correlation analysis was the strongest correlation in human TNBC with canine simple (r = 0.523) and ductal (r = 0.472) MGC. The existence of high transcriptomic correlation between canine MGC subtypes (ductal and simple) and human TNBC might be more important since TNBC has been highlighted in clinical and biomedical research due to its aggressive characteristics with poor prognosis. It has been known that the most common histological subtype of TNBC is invasive ductal carcinoma and their genetic profiles are shared by basal-like BC [39,40]. Thus, our results suggested that transcriptome signature of canine MGC and subtypes is able to represent the origin and characteristics of human BC. On the other hand, ER+-related human BC subtypes (ER+, ER+/HER2+) had few or no significant correlation with any canine MGC subtypes but tend to be shared by ductal and simple subtypes, respectively, in the given groups (Figure 4). However, this result, showing week correlation in ER+-related subtypes with canine MGTs, should be confirmed if it is influenced by spayed dogs. 

Since many studies have been performed in human breast cancers, we reviewed literature regarding human breast cancer and oncogenes to compare our findings to human studies. First, four out of 16 representative DEGs found in all three subtypes of canine MGTs have strong references in human cancer as biomarkers: CCL23, CXCL10, SFRP2, and FRZB. These genes are altered in at least four types of human cancers, including breast cancer [41,42,43]. Second, six genes, CHI3L1, CXCL8, FOXC2, SERPINE1, SFRP2, and TF, which are grouped within the highest enrichment GO term, “positive regulation of angiogenesis”, have been reported to play roles in various cancer processes, including breast cancer [44,45,46,47]. Moreover, 45 genes enriched in BP GO terms, ‘glycan biosynthesis and metabolism’ and ‘lipid metabolism’, may provide strong evidence that cellular metabolism is fundamentally altered in cancer tissue, and lipid metabolism may have crucial roles in cancer progression [48]. This survey confirms that dogs and dog MGCs are good animal models for human breast cancer study at the transcriptome level. 

We further investigated the biological roles of MGC subtype-enriched DEGs. KEGG pathway analysis using 211 up- and 306 down-regulated DEGs revealed that cancer signaling in the complex subtype was mainly triggered by Wnt-Frizzled LRP5/6 and GPCR signaling, whereas glycan biosynthesis and metabolism are strongly blocked through down-regulation of PPAR signaling, beginning with CD36-FABP. 

A total of 141 up- and 120 down-regulated DEGs were tested in the ductal subtype. Similar to the complex subtype, both glycan biosynthesis and lipid metabolism were down-regulated, but down-regulated retinol metabolism was found only in the ductal subtype. Although down-regulated biological processes were shared by two different subtypes, there were discrepancies in the list of up-regulated pathways between complex and ductal subtypes. KEGG pathways involved in cancer, such as cell adhesion, PIK3-Akt signaling, and ECM-receptor interaction, are enriched in the ductal subtype. Many ECM molecules have been associated with breast cancer development [49]. These discrepancies may partly come from differences in cellular origin, compositions of cell types and the cancer environment. 

Since only two pairs of specimens comprised the simple subtype, the number of identified DEGs was small (79 up- and 115 down- regulated). Focal adhesions as well as the Wnt and ECM-ITGB pathways were up-regulated. Interestingly, insulin signaling, including the FBP-1 gene, was the most highly enriched in down-regulated DEGs, but we know that down-regulation of FBP1 promotes tumor metastasis and indicates poor prognosis in other cancers [50]. If the results from the canine MGT subtype-enriched transcriptome profiles are validated in a large sample size, it will likely be helpful in developing cancer therapies for human breast cancer counterparts.

As previously stated, only a few aspects of PROMPT, a newly identified class of RNAs produced just upstream of the promoters of active protein-coding genes, have been characterized; due to being rapidly dumped by exosomes, their biological functions remain to be revealed [51,52]. We thus tested whether PROMPT expression can be detected in paired-end stranded total RNA sequencing data. First, we should note that the “PROMPT” we measured in this study might differ from the general term “PROMPT”. We used the term PROMPT since “promoter upstream transcripts” is exactly what we investigated in this study. However, many transcripts may not satisfy the criteria of the general term PROMPT in size or amount [27]. Furthermore, our measurements also have a few limitations to calculating accurate levels of transcript expression because small portions of non-coding RNAs including PROMPTs are annotated and characterized with their structures. We then measured all the sequence reads mapped upstream of the promoter region (−1500 bp~TSS) without consideration of RNA structures. It may not represent exact amounts of transcripts if the size is longer than 1500 bp or exon structures vary. 

In this study, we selected and showed two gene promoters upstream regions representing each correlation type (Figure 6A). Although negative correlation between genes and PROMPTs were stronger than positive correlations, positive correlations were more reliable because many genes with negative correlations were found as artifacts due to the low number of PROMPTs. Target-enriched high-throughput sequencing for short transcripts may be helpful for this type of analysis. Furthermore, comprehensive annotation with extensive transcriptome analysis in dogs is mandatory for comparative medicine and future study. In addition, diverse small-size non-coding RNAs, including micro RNA, which were not analyzed in this study due to the limitation of RNA isolation method but can be done by miRNA capturing in the future, might have very important roles in canine MGC as well.

Canine MGC has been proposed as a comparative model for spontaneous tumors of human BC due to their genetic, clinical, and biological similarities to human BCs. In addition, closely shared environmental conditions between dog and owner can be beneficial in an approach using epigenetic aberrations. Thus, studies for canine MGCs, counterparts of human BCs, can provide new clues for biomarker screening in human BCs (Figure 8C). We confirmed RNA sequencing data and validated three genes’ expression in additional sets of samples using quantitative real-time PCR. FN1 and BGN were targeted here due to their expression pattern being similarly up-regulated in human breast cancers. However, SCD was identified as a down-regulated gene in this study but is known to be up-regulated in human BCs. These results might represent similarities and discrepancies that exist between human BC and canine MGCs. 

## 4. Materials and Methods 

### 4.1. Specimens

This study was reviewed and approved by the Seoul National University Institutional Animal Care and Use Committee (IACUC# SNU-170602-1). Ten dogs diagnosed with mammary gland tumor were enrolled in this study. Mammary gland tumors and matching adjacent normal tissues were obtained by excisional surgery. Clinical features of eight dogs analyzed in the study are listed in Table S1. Eight pairs of specimens consisting of two simple-, 3 ductal-, and three complex-subtypes, from diverse breeds including Maltese, Dachshund, and Cocker Spaniel, were processed further for RNA sequencing. For total RNA sequencing, all tissue samples were immersed in RNAlater solution (Qiagen, Valencia, CA, USA) overnight at 4 °C, and stored at −80 °C after removal from solution. 

### 4.2. RNA Isolation and Total RNA Sequencing

Total RNA was extracted from mammary gland tumors and matched to normal tissues using the RNeasy Mini plus kit (Qiagen). Pulverization for sample homogenization was performed with liquid nitrogen before RNA isolation according to the manufacturer's instructions. The RNA quality was assessed by analysis of 18S and 28S rRNA band integrity on RNA 6000 Nano Kit (part # 5067-1511) using an Agilent Bioanalyzer (Agilent, Santa Clara, CA, USA). After ribosomal RNA (rRNA) depletion from 2 µg of total RNA, libraries were constructed using the TruSeq Stranded Total RNA Sample Preparation Kit (RS-122-9007) (Illumina, San Diego, CA, USA) according to the manufacturer’s guideline. The cDNA library quality was evaluated electrophoretically with an Agilent DNA 1000 Kit (part # 5067–1504) (Agilent). Subsequently, libraries were sequenced using Illumina HiSeq2500 that were set to rapid-run mode. Cluster generation, followed by 2 × 100 cycle sequencing reads, separated by paired-end turnaround, were performed on the instrument using HiSeq Rapid SBS Kit v2 (FC-402-4021) and HiSeq Rapid PE Cluster Kit v2 (PE-402-4002) (Illumina). Image analysis was performed using the HiSeq control Software version 2.2.58. The raw data were processed, and base-calling was performed using the standard Illumina pipeline (CASAVA version 1.8.2 and RTA version 1.18.64). A summary of statistics of the RNA sequencing data is listed in Table S2. 

### 4.3. Primary Analysis of RNA-seq Data (Mapping and Quantification)

Initially, transcript integrity was analyzed and transcript integrity number (TIN) was in Table S3. Reads were aligned with the dog reference genome (CanFam 3.1) using Hiset2 (ver.2.1.0) with cufflink option. Mapped reads were then assembled and counted using Cuffquant (ver. 2.2.1) and our GTF annotation file pre-built with additional transcripts information obtained from 13 different organs based on the Ensembl database (Canis lupus familiaris 3.1.91 gene set). Defaults were used for all other parameters. Numbers of transcripts identified in the study were listed in Table S4.

### 4.4. Differentially Expressed Gene (DEG) Analysis

For the differential gene expression analysis, three subtypes of MGC (simple, complex, and ductal) and three breeds, as well as all eight MGCs and matching normal tissues, were grouped and compared using Cuffdiff (ver.2.2.1). Genes with expression differences of 2-fold increases or decreases and *p* < 0.01 were evaluated as DEGs and were further analyzed. FPKM were extracted for all groups, and Plotly package in R was employed to visualize statistically significant changes among the comparisons (Table S5). Venn diagrams were created using Venny 2.1 (http://bioinfogp.cnb.csic.es/tools/venny/index.html). 

### 4.5. Correlation Analysis, Clustering and Principal Component Analysis (PCA)

FPKM values were extracted from a list of DEGs enriched in three subtypes of MGCs. All the FPKM values were log2 transformed to rank correlations among three subtypes of MGCs. Spearman rank correlation was calculated using Perseus ver.1.5.8.5 and visualized as Multiscatter plots in Maxquant software package (Max Planck Institute of Biochemistry, Munich, Germany). Z-scores were calculated from FPKM and further used for gene clustering. Clustering was performed with Kendall clustering method and the heat map was visualized using “pheatmap” in R package. PCA was performed by using ClustVis (https://biit.cs.ut.ee/clustvis/) [53].

### 4.6. Comparative Gene Expression Analysis among Four Subtypes of Human BC and Three Subtypes of Canine MGT

RNA-sequencing data for four molecular subtypes (HER2+, ER+, ER&HER2+, and TNBC) were retrieved from the project (PRJNA305054) in the National Center for Biotechnology Information (NCBI). The expression of orthologous genes, matched with subtype-specific DEGs and summarized in Table S6A, were compared in Spearman correlation and visualized in scatter plot using SPSS program. On the contrary, correlation in gene expression between canine MGC and human BC was computed using the list of genes in PAM50 and Oncotype DX (Table S6B). 

### 4.7. Pathway Enrichment Analysis and Gene Ontology (GO) Analysis

To better understand the biological significance of the identified DEGs, we performed GO, gene network analysis, and pathway enrichment analysis. GO was analyzed with overall MGTs-enriched and subtype-enriched DEGs using the web-based functional annotation tool DAVID 6.7 (https://david.ncifcrf.gov) and ClueGo, provide by Cytoscape App Store (apps.cytoscape.org). Three aspects, including biological process (BP), molecular function (MF), and cellular component (CC), were surveyed and the highest enrichment aspect, BP in this study, was documented in detail. GO terms and gene networks were visualized by ClueGo (cytoscape.org) [54]. For all GO and KEGG pathway analysis, *p* < 0.01 was considered as significant (Tables S7 and S8).

### 4.8. PROMPT Detection from Paired-End and Strand-Specific Total RNA Sequencing Data

To measure transcripts upstream of promoter regions, GTF file, reference annotation file was pre-processed to modify. Coordinates of each gene were switched to ranges from TSS of each gene to −1500 bp and aligned RNA reads were measured. Except for the modified GTF file, all the procedures were performed with the same protocol for genes. Using Microsoft Excel, correlation was calculated only in a list of DEGs which met the condition *p*-value < 0.01 and fold change > 2, with PROMPT of fold change > 2 (Table S9). 

### 4.9. RT-qPCR for RNA-seq Data Validation

Genomic DNA contamination-free RNA was isolated using the Qiagen RNeasy kit plus (Qiagen, CA). cDNA first-strand synthesis was achieved using OMMISCRIPT RT KIT (Qiagen). Primers for the top ten DEGs and A5B as the housekeeping gene were designed based on available sequences using GenBank (Table S10). Real-time PCR was performed on CFX96 Touch Real-Time PCR Detection System (Bio-Rad, Hercules, CA, USA) and relative gene expression to ATP5B were measured by the delta–delta CT method.

### 4.10. Statistics

For the GO and pathway analysis, ClueGO, which is a Cytoscape plugin using kappa statistics, was applied to perform a single-cluster analysis and comparison of clusters. Mann–Whitney U tests were applied to compare gene expression levels between MGCs and adjacent normal tissues in ROC curve. 

## 5. Conclusions

This study reports the comprehensive transcriptome profile of spontaneous canine MGCs and subtypes. Sets of differentially expressed genes in canine MGCs were determined from overall canine MGCs for each subtype. Many genes, but not all, listed in this study have been reportedly associated with human cancers including breast cancer. Three canine MGC subtypes then were matched to four human BC subtypes according to their transcriptome profiles. This study may represent the extant similarities between human BCs and canine MGCs. Thus, the current study provides new clues and clinical implications for better understanding of canine MGCs and their application to human BCs. Further validation using large sample numbers will reveal more general features, but our current study provides an important initial understanding of canine MGCs in different canine MGC subtypes. 

## Figures and Tables

**Table 1:** Top 5 up-/down-DEGs enriched in overall canine MGC and in three subtypes.

Group	Ensembl ID	Gene	log_10_(Fold Change)	−log_10_(*p*-Value)
Overall MGCs	ENSCAFG00000006046	COL6A5	6.06776	2.37161107
	ENSCAFG00000003825	MATN3	5.14522	4.301029996
	ENSCAFG00000024982	C4BPA	4.5425	2.288192771
	ENSCAFG00000000367	ENPP3	4.10668	4.301029996
	ENSCAFG00000017925	DLK1	4.02563	2.187086643
	ENSCAFG00000016014	KRT26	−11.005	2.381951903
	ENSCAFG00000011986	PLIN1	−10.1163	2.038578906
	ENSCAFG00000023806	KRT25	−10.001	2.869666232
	ENSCAFG00000017661	SERPINA12	−8.16046	2.361510743
	ENSCAFG00000017941	CYP1A2	−8.04964	2.677780705
Complex	ENSCAFG00000011534	ACAN	6.56988	2.004364805
	ENSCAFG00000012561	DMBT1	6.33988	4.301029996
	ENSCAFG00000004810	CXCL17	6.13628	2.26760624
	ENSCAFG00000012181	ACTC1	6.08066	3.698970004
	ENSCAFG00000002142	IL1RL1	5.92575	4.301029996
	ENSCAFG00000013694	ADIPOQ	−10.343	2.709965389
	ENSCAFG00000011986	PLIN1	−9.57036	2.314258261
	ENSCAFG00000005266	CIDEC	−9.47559	2.356547324
	ENSCAFG00000018828	CIDEA	−9.36545	3.397940009
	ENSCAFG00000001672	LEP	−8.18516	3.823908741
Ductal	ENSCAFG00000009820	NOS1	7.40628	2.769551079
	ENSCAFG00000005458	CLDN10	5.90219	2.853871964
	ENSCAFG00000014345	FN1	5.3764	4.301029996
	ENSCAFG00000002808	TMPRSS11B	5.3535	4.301029996
	ENSCAFG00000008948	LYZF2	5.3447	2.744727495
	ENSCAFG00000023094	MYH3	−10.6876	2.002176919
	ENSCAFG00000018070	DSC1	−10.3628	2.920818754
	ENSCAFG00000023806	KRT25	−10.0193	4.301029996
	ENSCAFG00000015475	DES	−9.0685	2.431798276
	ENSCAFG00000011103	NRAP	−8.30778	4.301029996
Simple	ENSCAFG00000006046	COL6A5	9.15426	2.361510743
	ENSCAFG00000013863	CEMIP	7.65997	2.167491087
	ENSCAFG00000000834	TNFRSF11B	6.89898	3.397940009
	ENSCAFG00000020033	CLEC3A	6.45938	2.200659451
	ENSCAFG00000003825	MATN3	6.0326	2.37675071
	ENSCAFG00000011103	NRAP	−10.1506	2.099632871
	ENSCAFG00000014281	PYGM	−8.87425	2.296708622
	ENSCAFG00000028609	TNNC2	−8.60343	2.019996628
	ENSCAFG00000008950	LALBA	−8.18348	4.301029996
	ENSCAFG00000014842	MYOZ1	−7.72731	2.164309429

**Table 2:** Gene ontology (GO) terms biological processes (BP) of up- and down-regulated DEGs in canine MGCs.

Up-Regulated DEGs					
GO groups	GO ID	GO Term	% Assoc. Genes	No. Genes	Associated Genes Found
0	:1904018	positive regulation of vasculature development	4.58	6	[CHI3L1, CXCL8, FOXC2, SERPINE1, SFRP2, TF]
	:0045766	positive regulation of angiogenesis	4.8	6	[CHI3L1, CXCL8, FOXC2, SERPINE1, SFRP2, TF]
	:0031638	zymogen activation	4.07	5	[PLAU, S100A8, SERPINE1, SERPINE2, TF]
	:0033627	cell adhesion mediated by integrin	5.26	4	[FOXC2, PLAU, SERPINE1, SFRP2]
	:0033628	regulation of cell adhesion mediated by integrin	7.27	4	[FOXC2, PLAU, SERPINE1, SFRP2]
	:1903318	negative regulation of protein maturation	10.34	3	[C4BPA, SERPINE1, SERPINE2]
	:0010955	negative regulation of protein processing	10.34	3	[C4BPA, SERPINE1, SERPINE2]
	:0031639	plasminogen activation	17.65	3	[PLAU, SERPINE1, SERPINE2]
1	:0097529	myeloid leukocyte migration	4.19	7	[CCL8, CMKLR1, CXCL10, CXCL8, S100A8, SERPINE1, SPP1]
	:0097530	granulocyte migration	4.17	5	[CCL8, CMKLR1, CXCL8, S100A8, SPP1]
	:0071222	cellular response to lipopolysaccharide	4.26	6	[CD80, CD86, CXCL10, CXCL8, SERPINE1, TNIP3]
	:0002690	positive regulation of leukocyte chemotaxis	5.26	4	[CMKLR1, CXCL10, CXCL8, SERPINE1]
	:0070098	chemokine-mediated signaling pathway	4.94	4	[CCL8, CMKLR1, CXCL10, CXCL8]
	:0071621	granulocyte chemotaxis	4.39	5	[CCL8, CMKLR1, CXCL8, S100A8, SPP1]
2	:0051607	defense response to virus	4.7	7	[CD86, CXCL10, ITGAX, PTPRC, RSAD2, SAMHD1, TLR7]
	:0002224	toll-like receptor signaling pathway	5.13	4	[CD86, RSAD2, TLR7, TNIP3]
3	:0050654	chondroitin sulfate proteoglycan metabolic process	8.82	3	[BGN, CHST11, NDNF]
	:0030204	chondroitin sulfate metabolic process	11.11	3	[BGN, CHST11, NDNF]
4	:0002456	T cell-mediated immunity	4.05	3	[P2RX7, PTPRC, RSAD2]
5	:0045124	regulation of bone resorption	9.38	3	[P2RX7, TF, TFRC]
6	:1901292	nucleoside phosphate catabolic process	4.11	3	[ENPP3, P2RX7, SAMHD1]
7	:0034405	response to fluid shear stress	9.09	3	[COX-2, P2RX7, SPP1]
**Down-Regulated DEGs**					
0	:0086036	regulation of cardiac muscle cell membrane potential	27.27	3	[ANK2, FXYD1, TRDN]
	:1903513	endoplasmic reticulum to cytosol transport	9.43	5	[ANK2, DHRS7C, DMD, RYR1, TRDN]
	:1903514	calcium ion transport from endoplasmic reticulum to cytosol	11.11	5	[ANK2, DHRS7C, DMD, RYR1, TRDN]
	:0070296	sarcoplasmic reticulum calcium ion transport	10.64	5	[ANK2, DHRS7C, DMD, RYR1, TRDN]
	:0014808	release of sequestered calcium ion into cytosol by sarcoplasmic reticulum	11.11	5	[ANK2, DHRS7C, DMD, RYR1, TRDN]
1	:0055088	lipid homeostasis	7.37	7	[ANGPTL4, DGAT2, EPHX2, GPAM, LCAT, LPL, RORA]
	:0055090	acylglycerol homeostasis	13.79	4	[ANGPTL4, DGAT2, LPL, RORA]
	:0070328	triglyceride homeostasis	14.81	4	[ANGPTL4, DGAT2, LPL, RORA]
2	:0009755	hormone-mediated signaling pathway	5.56	7	[ACSL1, AR, BMP4, ESR1, PPARG, PRLR, RORA]
	:0060850	regulation of transcription involved in cell fate commitment	17.39	4	[BMP4, PPARG, PROX1, RORA]
3	:0006638	neutral lipid metabolic process	6.32	6	[DGAT2, GPAM, LIPE, LPIN1, SERPINA12, TNXB]
	:0006639	acylglycerol metabolic process	6.45	6	[DGAT2, GPAM, LIPE, LPIN1, SERPINA12, TNXB]
4	:0055001	muscle cell development	4.12	8	[ANK2, BMP4, COL14A1, CSRP3, DMD, PROX1, RYR1, TTN]

## Supplementary Materials

The following are available online at http://www.mdpi.com/2072-6694/10/9/317/s1. Supplementary Table S1, Specimens. Supplementary Table S2, RNA sequencing statistics. Supplementary Table S3, Transcript integrity analysis. Supplementary Table S4, Numbers of transcripts identified. Supplementary Table S5, List of DEGs up-and down-regulated in MGCs. Supplementary Table S6, Correlation analysis of canine MGC subtypes and human BC subtypes. Supplementary Table S7, GO analysis of MGC subtype-enriched DEGs. Supplementary Table S8, Up- and Down- regulated gene list. Supplementary Table S9, Correlation between DEGs and PROMPTs. Supplementary Table S10, Primers for validation. Supplementary Figure S1, KEGG pathways analysis integrated DEG data.
